# Surface grafting of polymeric catheters and stents to prevent biofilm formation of pathogenic bacteria

**DOI:** 10.1186/s43141-023-00545-2

**Published:** 2023-09-14

**Authors:** Reham G. Elfarargy, Mohamed Sedki, Farag A. Samhan, Rabeay Y. A. Hassan, Ibrahim M. El-Sherbiny

**Affiliations:** 1 Center for Materials Science, Zewail City of Science and Technology, 6Th October City, Giza, 12578 Egypt; 2 Water Pollution Research Department, National Research Centre (NRC), Giza, Egypt

**Keywords:** Electro-deposition, Polymeric stents and catheter biomaterials, Tecothane chemical grafting, Biofilm inhibition, Bio-electrochemical systems

## Abstract

**Background:**

Tecothane (medical grade of polyurethane) is strongly involved in the fabrication of metallic and polymeric-based medical devices (e.g., catheters and stents) as they can withstand cardiac cycle-related forces without deforming or failing, and they can mimic tissue behavior. The main problem is microbial contamination and formation of pathogenic biofilms on such solid surfaces within the human body. Accordingly, our hypothesis is the coating of tecothane outer surfaces with antibacterial agents through the electro-deposition or chemical grafting of anti-biofilm agents onto the stent and catheter surfaces.

**Results:**

Tecothane is grafted with itaconic acid for cross-linking the polyethyleneimine (PEI) as the protective-active layer. Accordingly, the grafting of poly-itaconic acid onto the Tecothane was achieved by three different methods: wet-chemical approach, electro-polymerization, or by using plasma treatment. The successful modifications were verified using Fourier Transform Infrared (FTIR) spectroscopy, grafting percentage calculations, electrochemical, and microscopic monitoring of biofilm formation. The grafting efficiency of itaconic acid was over 3.2% (w/w) at 60 ℃ after 6 h of the catheter chemical modification. Bio-electrochemical signals of biofilms have been seriously reduced after chemical modification because of the inhibition of biofilm formation (for both *Pseudomonas aeruginosa* and *Staphylococcus aureus*) over a period of 9 days.

**Conclusion:**

Chemical functionalization of the polyurethane materials with the antimicrobial and anti-biofilm agents led to a significant decrease in the formation of pathogenic biofilms. This promising proof-concept will open the door to explore further surface protection with potential anti-biofilm agents providing better and sustainable productions of stents and catheters biomaterials.

**Graphical Abstract:**

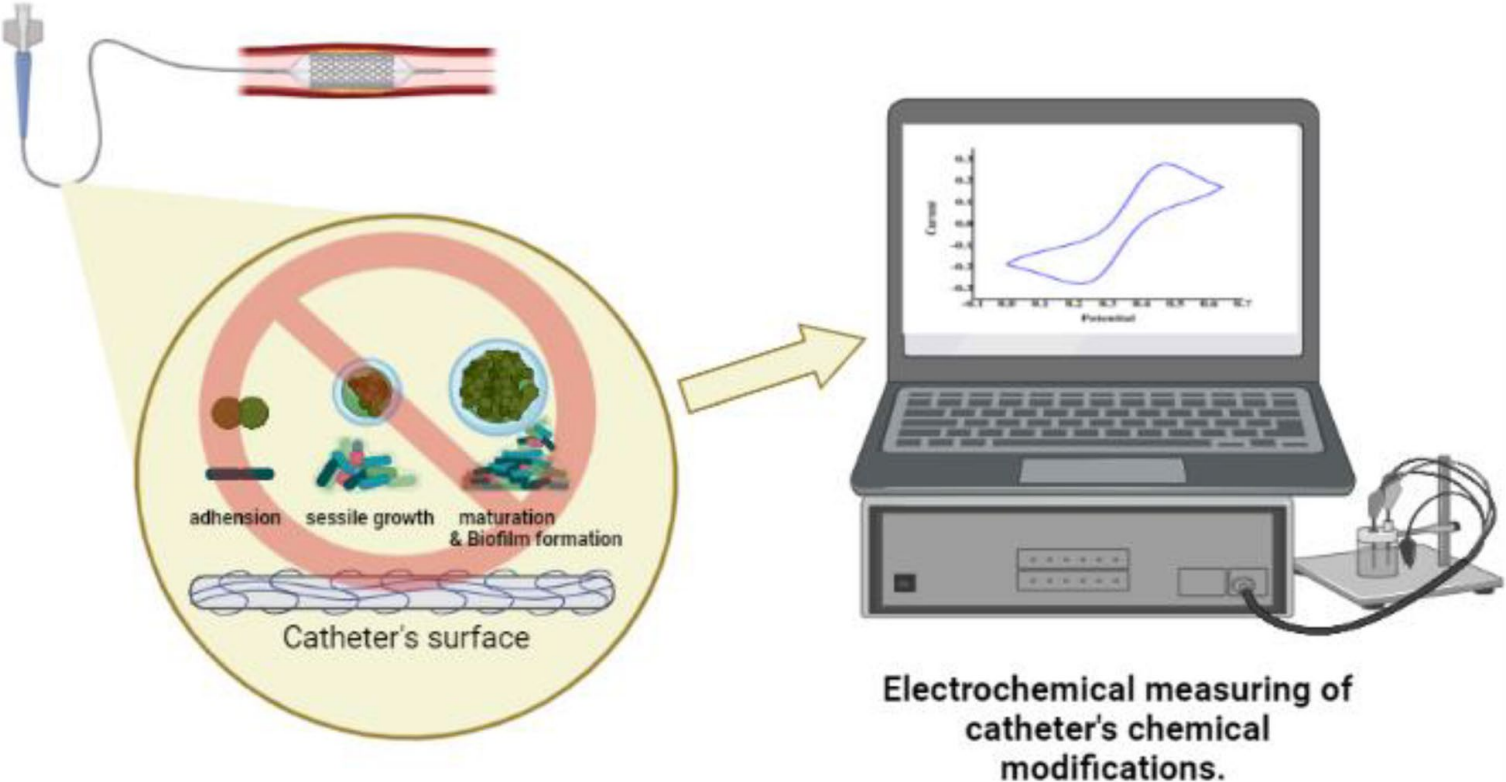

## Background

Biomedical devices such as central venous catheters, prostheses, or urinary catheters improved patients' healthcare and advanced medicine. However, there is a variety of opportunistic pathogens that cause implant infections. Accordingly, biomedical device-associated infections are accounted for a large proportion of hospital-acquired infections [1, 2]. Statistically, implant-associated infections are responsible for about 26% of all healthcare-associated infections in the USA [3]. Without the insertion of implants, an infection triggered by opportunistic pathogens is spontaneously cleared by host immune defenses. Nevertheless, in devices-associated infections, the foreign body activates a local tissue response, which includes a formation of granulation tissue, acute and chronic inflammation, and, eventually, fibrous encapsulation [4]. Bacteria adhere to the surface of biomaterial and subsequently forming of biofilm that either protects the bacterial cells from any threat from the host's immune system or provided antibiotics. The classic approach of biofilm formation, which applies to *Staphylococcus aureus* and to Gram-negative bacteria such as *Pseudomonas aeruginosa*, comprises several phases: bacterial adhesion to the host materials, microcolony formation because of cellular aggregation, exopolysaccharide (EPS) construction, and towers formation. Dispersion of biofilms occurs when colonized bacteria return to a planktonic state [5, 6].

Regarding the material used, commonly Tecothane (TC) catheters (medical grade of polyurethane) have been used in many medical applications due to their chemical stability, strong mechanical and physical properties as well as its high biocompatibility. However, Tecothane-based catheters are highly susceptible to microbial infections and show a higher thrombogenicity, compared to other types of catheters [6]. Among a long list of Tecothane-related bloodstream bacterial infections, *P. aeruginosa* and *S. aureus* are the main causes for lethal complications that arise from intravenous catheterization, especially central venous catheters (CVCs). They are considered as one of the essential sources of septicemia and bacteremia [7]. Accordingly, it is a necessity to do surgical replacement for the biofilm-infected catheter in order to avoid spreading a chronic infection to other internal organs. Besides surgical risks associated with replacing infected catheters, there is no assurance that pathogens will not re-colonize upon the new device made of the same material [8]. Many studies have been devoted to preventing biofilm formation on catheters or other bio-implanted materials, where impregnating catheter surfaces with broad-spectrum antibiotics were suggested to elute the bacteria colonization [9, 10]. However, this approach increased the proportion of antibiotic-resistant microbes, while the adhered bacteria on the implanted material showed higher levels of antibiotic resistance than that obtained by the bacterial suspension [11]. Linear or branched structures Polyethylenimine (PEI), the abundant surface amine groups, have been intensively exploited in biomedical imaging, antimicrobial preparation, gene therapy, and in the controlled drug delivery systems [12]. Therefore, the PEI has been frequently used to functionalize biomaterials for different biomedical applications; for example, it has been used as a carrier system to encapsulate anticancer drugs, for tissue engineering and gene therapy applications. Moreover, the PEI has been used as an antibacterial and anti-biofilm agent due to the electrostatic interaction between positively charged PEI and the negatively charged bacterial cell walls [13–16]. The primary cause of PEI toxicity towards human cell its strong positive charge, which causes PEI to interact strongly with cell surfaces and cause necrosis to them.

In our study, Tecothane polymer is linked with PEI through Itaconic acid that eradicates the toxicity of the PEI by reacting with the positive charge of primary and secondary amines.

Antimicrobial activity of the PEI against common bacteria and yeasts, regarding planktonic cells and biofilm, along with understanding its antimicrobial mechanism of action, were determined [16–18]. Among the list of screening microorganisms, *Enterococcus faecalis*, *Staphylococcus aureus*, *Escherichia coli*, and *Candida albicans* strains were susceptible to the PEI actions. The effect of PEI was evaluated against the cell adhesion, biofilm formation, and biofilm disaggregation. In order to understand PEI cellular effects, flow cytometric analysis was performed with different fluorescents [16]. From the diagnostic point of view, it is not only difficult to treat the formed biofilms, but also it is difficult to detect the viability as well as the antibiotic susceptibility. In this regard, there are many methods for the detection and assessment of biofilm growth, such as plate count and viable cell enumeration, light microscopy, fluorescent microscopy and staining, and scanning electron microscopy (SEM). These techniques are time-consuming and limited the inaccurate determination of biofilm growth. Recently, bio-electrochemical approaches were used to evaluate the growth of active biofilms on solid substrates [19]. Conceptually, microorganisms have the ability to transfer electrons from/to solid conductive materials resulting in an electric current generation [20, 21]. The height or intensity of such electric current is proportional to the number of viable cells involved in the electron transfer process. Thus, tracking the intensity of the generated electric current is applied for the online monitoring of biofilm formation [22].

In this work, our hypothesis is the coating of tecothane outer surfaces with antibacterial agents through the electro-deposition or chemical grafting of anti-biofilm agents onto the stent and catheter surfaces. Thus, the implant-associated infections problem is tackled through the chemical grafting of the Tecothane surface with anti-bacterial agents without affecting the distinguishable properties of that polymer. Accordingly, catheters made of Tecothane were grafted with polyethyleneimine (PEI) through itaconic acid (IT) as a linker. Moreover, bio-electrochemical systems for testing the bacterial activity and online monitoring the biofilm progression on the catheters surfaces (treated or untreated) were implemented. In addition, SEM imaging was utilized to monitor bacterial growth on the material surface, as a confirmatory experiment.

## Methods

### Preparation of Tecothane casted film and grafting with Itaconic acid

Following the method reported by Mehlika Pulat et al. [23], 4.0 g of Tecothane powder was dissolved in 50 mL tetrahydrofuran (THF) and left for 24 h to complete dissolution. The tecothane solution was poured in a glass dish and left to evaporate the solvent for 24 h at room temperature to form a homogenous layer. This layer was removed and cut to equal pieces (1.0 cm 2.0), then washed twice with ethanol using an ultrasonic bath for 10 min to remove any residuals. The layers were left to dry for another 24 h at room temperature. For initiating polymerization and grafting with the Itaconic acid, chemical grafting 1.0 g of Itaconic acid was dissolved in 10 mL ethanol (99%). Then each piece of Tecothane was immersed into the solution of Itaconic acid in a separate beaker. Then, 50 mg of tin (II) chloride was added as a catalyst. The reaction in each beaker was resumed under nitrogen purge for different time intervals (4.0, 6.0, 8.0, 24, 48, and 72 h) and at different temperatures (40, 60, and 80 ºC). The grafted Tecothane samples were washed twice with ethanol using ultrasonic bath for 10 min to remove the unreacted itaconic molecules. Finally, samples were left to dry at room temperature for 24 h. For initiating polymerization and grafting with the Itaconic acid, two different methods were implemented and their efficiencies were compared to each other. The grafting methods are discussed below:

### Chemical grafting

1.0 g of Itaconic acid was dissolved in 10 mL ethanol (99%). Then each piece of Tecothane was immersed into the solution of Itaconic acid in a separate beaker. Then, 50 mg of tin (II) chloride was added as a catalyst [24, 25]. The reaction in each beaker was resumed under nitrogen purge for different time intervals (4.0, 6.0, 8.0, 24, 48, and 72 h) and at different temperatures (40, 60, and 80 ºC). The grafted Tecothane samples were washed twice with ethanol using ultrasonic bath for 10 min to remove the unreacted itaconic molecules. Finally, samples were left to dry at room temperature for 24 h before recording their weights.

### Electro-polymerization (electro-deposition) using cyclic voltammetry (CV)

Cyclic voltammetry (CV) was used for performing electrochemical polymerization and electro-deposition of itaconic acid on the Tecothane layers where small Tecothane pieces were used as a working electrode in the voltammetric signal. Multiple voltammetric scans (20 cycles) were conducted in the itaconic acid solution (10%) to form an electro-polymerized film of itaconic acid directly on the Tecothane surface. CV scans were applied in the potential range of − 0.3 V to 1.0 V vs Ag/AgCl (in 3 M KCl).

In all the grafting methods, percentage of grafting (% GE) was evaluated according to the following equation:

$$\mathrm{\%\;GE }= 100\;(\mathrm{W}2-\mathrm{W}1)/\mathrm{W}3$$

where W1, W2, and W3 are the weights of TC, (TC + IT) copolymer, IT monomers, respectively.

### Functionalization of the grafted surface with polyethyleneimine (PEI)

As coupling agent, EDC (*N*-(3-dimethylaminopropyl)-*N*’-ethylcarbodiimide hydrochloride) and NHS (*N*-hydroxysuccinimide) were used. Accordingly, the EDC/NSH crosslinking was used for the surface functionalization whereas the grafted surface of Itaconic-Tecothane was immersed in aqueous NSH solution (46 mg/mL) for 1.0 h under stirring. Consequently, the surface was transferred to a solution of the EDC (30 mg/mL) for 10 min. Eventually, the surface was removed from the EDC, washed and inserted into the solution of the PEI for 24 h.

**Scheme 1:**
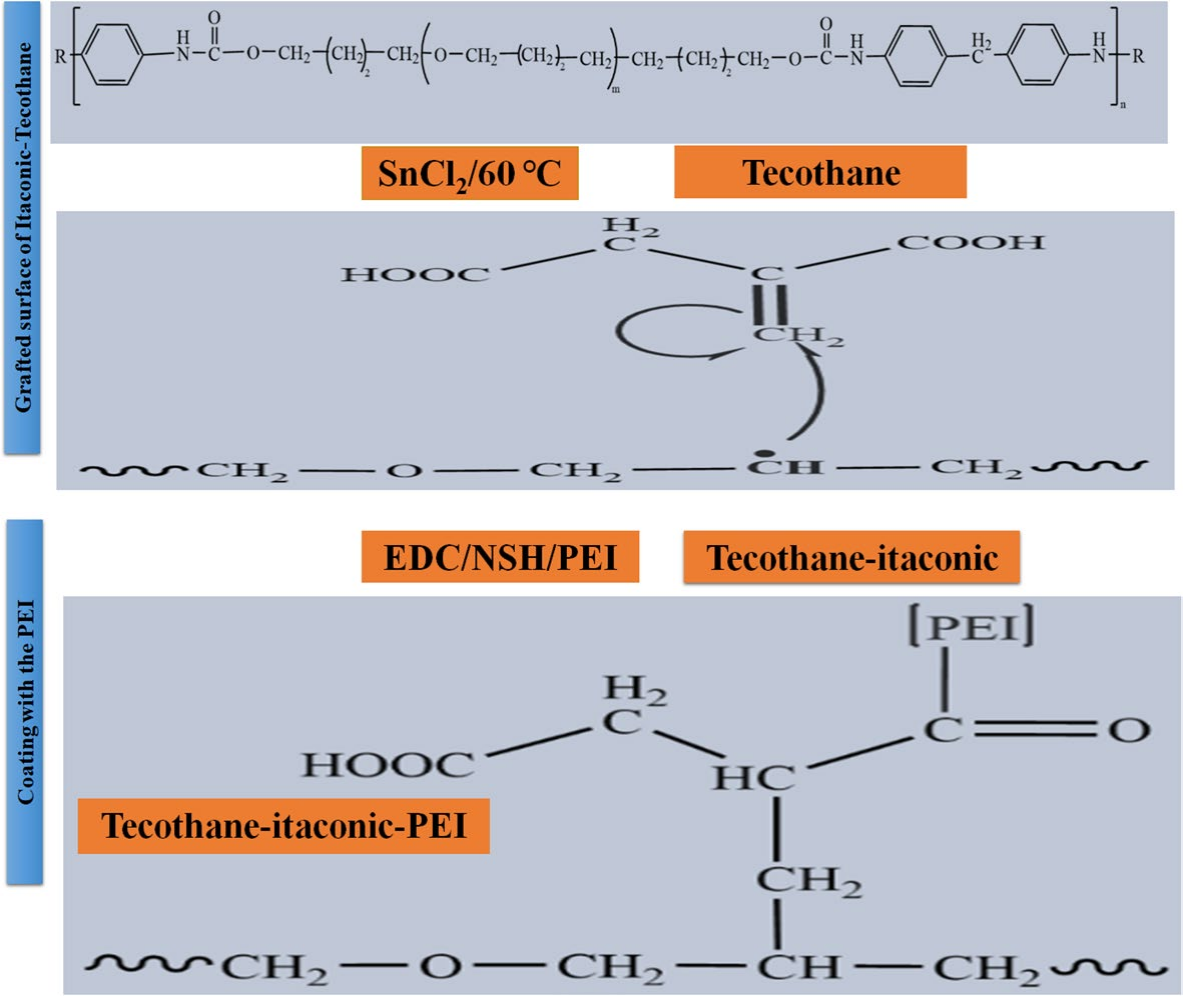
Steps of chemical grafting of Tecothane (Tc) surface with the *polyethyleneimine* (PEI)

Structural scheme (Scheme 1) is drawn for demonstration the preparation steps.

### Antibacterial effect of polyethyleneimine-coated surfaces

Tow bacterial strains; *Pseudomonas aeruginosa* (ATCC 10145) as Gram-negative and *Staphylococcus aureus* (ATCC 43300) as Gram-positive were included in this experiment. The bacterial strains were grown overnight in 50 mL aliquots of trypticase soy broth at 37 ºC, then their cell pellets were collected through the centrifugation at 2700 rpm for 5.0 min. The supernatant was decanted and the sediment was washed twice using a buffered saline solution containing/100 mL (KH_2_PO_4_ 0.18 g, Na_2_HPO_4_ 0.81 g) at pH 7.4. The cell count of each strain suspension was diluted to reach 1.0 × 10^7^ colony forming unit per milliliter (CFU/mL).

For testing the antibacterial effect of the grafted solid surfaces against two bacterial strains (*P. aeruginosa* and *S. aureus*), bacterial suspension (OD_600_ = 0.2) was prepared in tryptone soya broth (TSB) medium and the polymeric surface treated and untreated with the PEI was incubated for 45 min at 37 ºC. Then, 10 µL of water-soluble tetrazolium salt (WST) reagent were added and incubated for 30 min. The bacterial activity is inducing a yellow color (the reduced form of the WST reagent) through their metabolic reactions. The yellow color intensity was measured spectrophotometrically at 450 nm. PEI’s antibacterial effect was calculated by referring to the untreated cell suspensions.

### Bio-electrochemical assessment of biofilm growth

In bio-electrochemical systems, two groups of modified electrodes surface with bare Tecothane, Tecothane-Itaconic, or Tecothane-Itaconic-PEI were incubated with individual suspensions of fresh cultured *P. aeruginosa* and *S. aureus*. After 2 weeks from the incubation, electrochemical signals were measured using cyclic voltammetry to assess the growth of bacterial biofilms on each surface.

## Results

Polyurethanes are being applied in medical devices because they have the capability to tolerate contractile forces that originate during the cardiac cycle without undergoing plastic deformation or failure, and the capability to imitate the behaviors of different tissues. Chemical grafting of polyurethane surfaces was conducted to fight the formation of biofilms. Due to the high chemical stability of polyurethane, formation of a covalent bond between Tecothane and Itaconic acid was conducted through the activation of free radicals that were initiated by Tin chloride. Then the EDC/NSH reaction was used to activate the carboxyl groups of itaconic acid to ease the formation of amide bonds between Tecothane-Itaconic and PEI. Detailed steps of TC grafting with IT and PEI are shown in Scheme 1.

### Characterization of the grafted surface

**Fig. 1:**
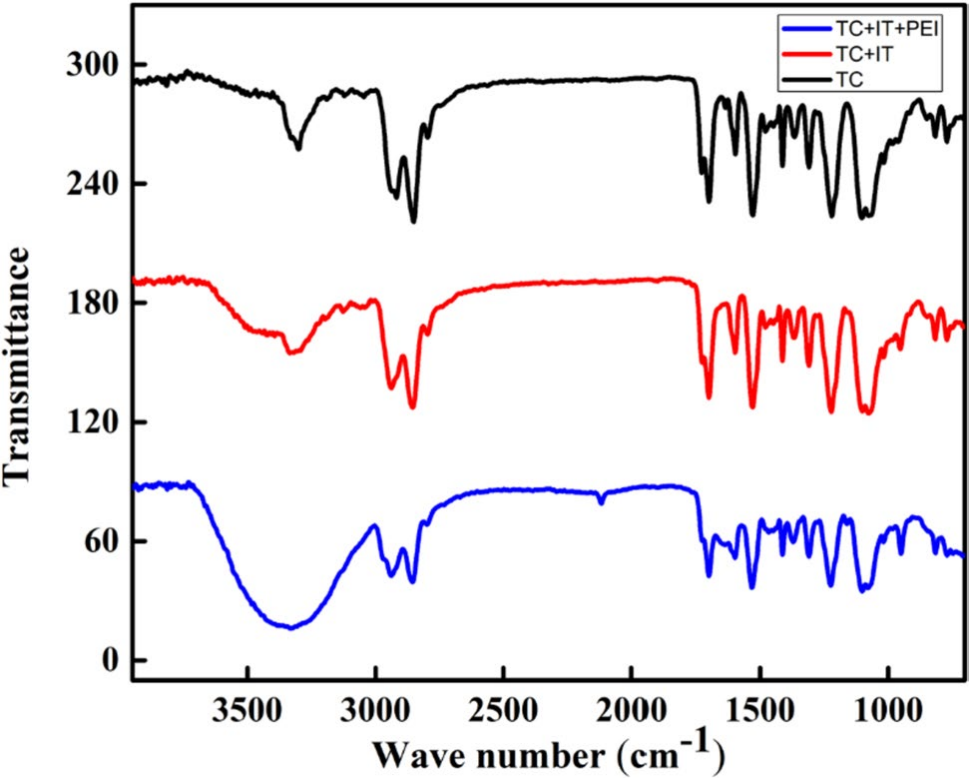
FTIR spectra of the of TC, TC + IT, and TC + IT + PEI

As shown in Fig. 1, FTIR spectra were obtained for each of the functionalization steps to show the different function groups added to the surface of Tecothane (TC) because of grafting with Itaconic acid (IT) which is then followed by the covalent binding with the polyethyleneimine (PEI). The IR-peak appears at 1638 cm^−1^ indicate the primary amide I: C = O stretching in Tecothane. This peak disappeared in the graph of TC + IT that emphasizes the formation of a bond between NH in tecothane and itaconic acid. Peak at 1649 cm^−1^ is attributed to the starching of the C = O, while the other band appeared at 3318 cm^−1^ is referring to the N–H in the TC + IT + PEI indicating the formation of the primary amide as a result of the reaction between TC + IT and PEI in forming an amide bond between them. The peak at 3305 cm^−1^ in the three graphs is indicating the O–H group. It is sharp in TC + IT because of the O–H from Tecothane and another broad peak at 3318 cm^−1^ indicating the presence of a carboxylic group in itaconic acid. In TC + IT + PEI.

### Measuring the grafting efficiency

**Fig. 2:**
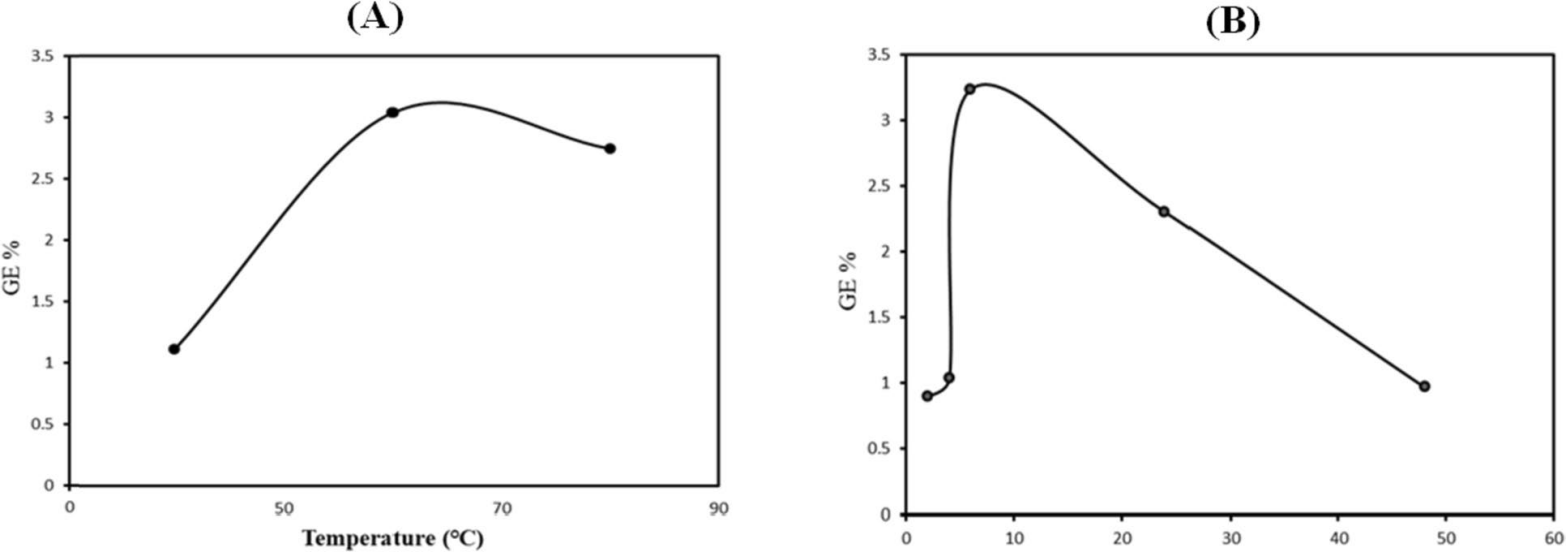
Effect of polymerization temperature (**A**), and polymerization time (**B**) on the grafting efficiency

Since the chemical grafting was performed at different conditions (i.e., changing the time of polymerization or temperature), the yield percentage of each condition was evaluated. Regarding the temperature effect on the grafting efficiency (GE), the GE % increased with increasing the temperature and it reached the maximum value at 60 ℃, as can be depicted from Fig. 2A. After that, a decrease in grafting yield was recorded. This increase in the grafting efficiency is attributed to increase of diffusion as well as mobility of itaconic acid. The decrease in the grafting efficiency with temperatures above 60 ℃ is assigned to the termination of polymerization, forming of homopolymer and denaturation of polymers [26].

In terms of the polymerization time effects, at fixed temperature (60 ℃), different time intervals were tested. The grafting efficiency was found to be dependent on the increase of the reaction time until reaching 6 h (Fig. 2B).

### Morphological analysis using scanning electron microscopy (SEM)

**Fig. 3:**
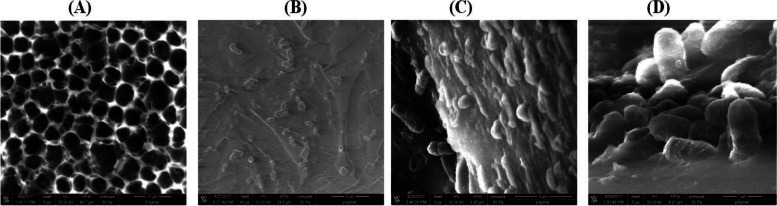
SEM images of untreated techothane solid surfaces before the formation of bacterial biofilm (**A**, **B**), and after the biofilm progression (**C**, **D**)

Tecothane is an excellent polymer with many advantages such as its mechanical strength, flexibility as well as biocompatibility that makes it potential candidate for fabrication of implantable material [27]. However, Tecothane is considered a suitable environment for biofilm formation. Figure 3 showed scanning electron microscopy images of the untreated surface of Tecothane, as in Fig. 3A. By zooming-in in one of the pore-like structures, it appears as a flat surface as in Fig. 3B. After 3 days of exposure to *P. aeruginosa*, the biofilm started to grow on TC’s layer as shown in Fig. 3B. The biofilm grew dramatically through time until a dense mature biofilm was formed after 10 days as shown in Fig. 3C, D.

### Evaluating the rate of biofilm formation using cyclic voltammetry

**Fig. 4:**
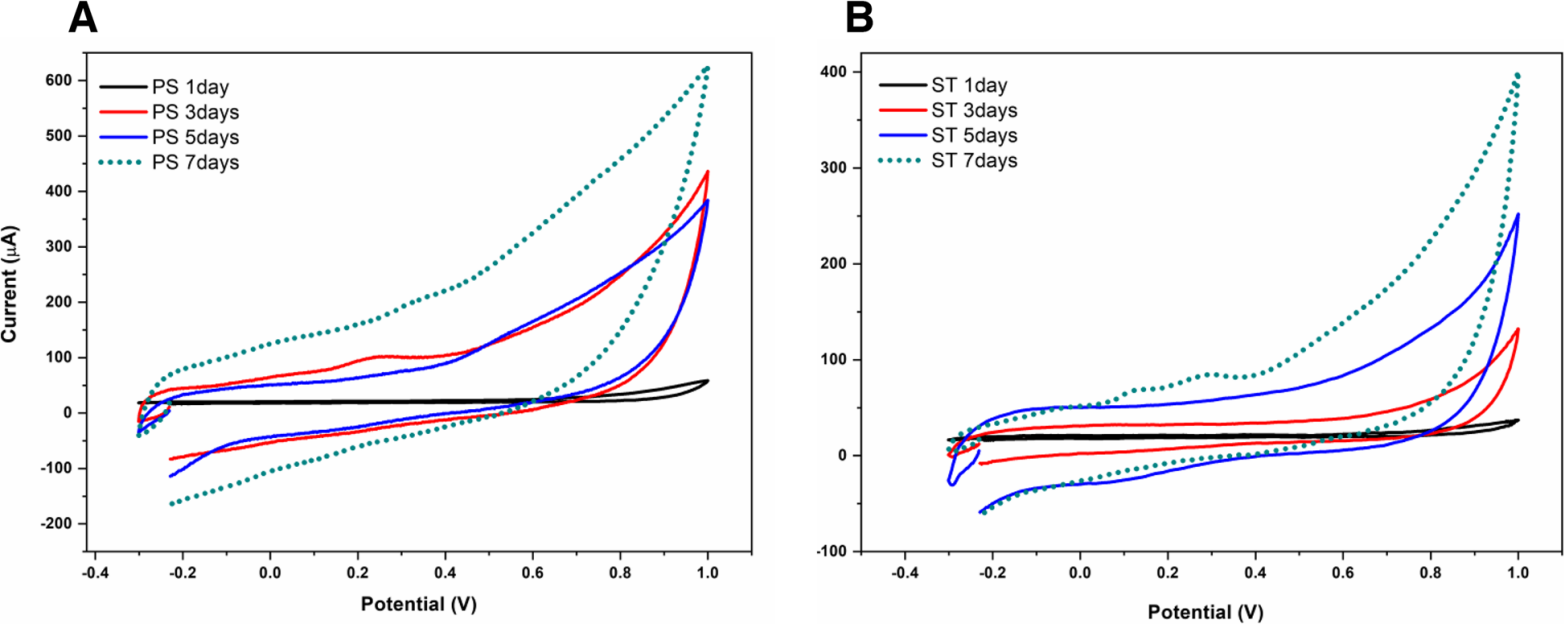
**A** Monitoring the biofilm formation of *P. aeruginosa* (PS) on the surface of a Tecothane-based working electrode. The cyclic voltammetric measurements were conducted at the scan rate of 50 mV/s and potential range from 0.3 to 1.0 V vs the Ag/AgCl. **B** Monitoring the biofilm formation of *S. aureus* (ST) on the surface of a Tecothane-based working electrode. The cyclic voltammetric measurements were conducted at the scan rate of 50 mV/s and potential range from 0.3 to 1.0 V vs the Ag/AgCl

The online monitoring of the biofilm progression and the effect of material grafting was conducted with the cyclic voltammetry [28], where the synthesized materials were placed on the outer layer of the working electrode that was incubated with bacterial cultures for a long duration. As shown in Fig. 4A, Faradic current with 136 µA was generated after the growing and adhering of *P. aeruginosa* cells onto the Techothane surfaces indicating the formation of a mature biofilm. Similarly, the Faradic current of 67 µA was generated after the adherence of *S. aureus*. The results of cyclic voltammetry are consistent with the SEM images. The bacteria grow vigorously on the untreated Tecothane surfaces. Accordingly, the cyclic voltammetry of the different modified materials incubated with bacterial cultures for 9 days were measured and the height of faradic currents were compared with the untreated Techothane surfaces. As a result, a strong inhibition in the electric current was obtained when the TC-IT-PEI was used as a working electrode (as shown in Table 1).

**Table 1:** This table shows the bio-electrochemical signals of the biofilm formation of *P. aeruginosa* (PS), and *S. aureus* (ST) on the coated catheter surfaces (TC polymer and the modified ones (TC + IT, TC + IT + PEI)

Material coating	*P. aeruginosa*	*S. aureus*
TC	180 *µ*A	122 *µ*A
TC + IT	49 *µ*A	66 *µ*A
TC + IT + PEI	49 *µ*A	36 *µ*A

## Discussion

Polyurethanes (PUs) have been formulated and modified to provide the high mechanical strength, high abrasion resistance, good biocompatibility, flexural endurance, and processing versatility over a wide range of other features [29]. These characteristics are very important in supporting new biomedical industries such as intra-aortic balloon pumps, artificial hearts, surgical drains, catheter tubing, feeding tubes, non-allergenic gloves, dialysis devices, wound dressings and more applications in the manufacture and designing of cardiovascular devices [30]. However, bacterial adhesion and biofilm formation on the surface of biomedical devices are the major concerns. Therefore, grafting of the surface of Tecothane with antimicrobial polymers, such as the polyethyleneimine, through the Itaconic acid as a chemical linker was designed. In this stud, decreasing the implant-associated infectious has been achieved through the chemical grafting of the Tecothane surface with anti-bacterial agents without affecting the distinguishable properties of that polymer. In this regards, the catheters made of Tecothane were chemically grafted with polyethyleneimine (PEI) through itaconic acid (IT) as a linker. The chemical grafting was carried out by different methods in order to define the affecting factors, as well as enhancing the grafting efficacy. The grafting efficiency was found to be dependent on the reaction time until reaching 6 h and then a decrease in the efficiency yields was observed. Two main reasons could be addressed to explain the decrease of grafting yield after exceeding this reaction time. First, the formation of saturated active sites and limiting the availability of free radicals or the vacant positions for PEI binding. Second, the polymers can be denatured because of being heated for a long time in ethanol, which is the reaction medium. As a model microorganism for testing the anti-biofilm characters of the PEI-treated techothane surfaces, *P. aeruginosa* as well as *S. aureus* were exposed to treated and untreated PEI-techothane surfaces, while their biofilm formation was evaluated using bio-electrochemical systems. Cyclic voltammetry could be used for real time measuring the cell viability, along with the metabolic activity, microbial adherence, colonization and biofilm formation. Thus, online monitoring of the biofilm progression and the effect of material grafting was conducted where the synthesized materials were placed on the outer layer of the working electrode which was incubated with bacterial cultures for a long duration. Thus, the bio-electrochemical signals of the selected two bacterial strains were recorded over along time of incubation. The collected electrical current is representing the rate of biofilm formation on the techothane materials [31]. As shown in Table 1, the electrical current was significantly dropped when the techothane surfaces were coated with the potential anti-biofilm agent.

## Conclusion

To overcome the bacterial growth and biofilm formation on Tecothane, and maintain its excellent characteristics, Tecothane polymer was grafted with itaconic acid as a linker then grafted with the antibacterial polymer (PEI). For monitoring of biofilm formation on Tecothane surface, SEM imaging and cyclic voltammetry were applied. SEM images have shown a strong biofilm formation on the un-grafted Tecothane surface after incubation with bacteria for 9 days, which emphasizes on the necessity and importance of surface modification of Tecothane with antimicrobial agents. From the voltametric measurements, there was a significant reduction of biofilm formation after surface modification with IT and PEI. This study reports different techniques to graft Tecothane with itaconic acid that opens the door for further modification and enhancing Tectophase’s properties.

## Data Availability

The datasets used in this study are available upon request through the corresponding authors.
